# Tracking and Predicting Depressive Symptoms of Adolescents Using Smartphone-Based Self-Reports, Parental Evaluations, and Passive Phone Sensor Data: Development and Usability Study

**DOI:** 10.2196/14045

**Published:** 2020-01-24

**Authors:** Jian Cao, Anh Lan Truong, Sophia Banu, Asim A Shah, Ashutosh Sabharwal, Nidal Moukaddam

**Affiliations:** 1 Electrical and Computer Engineering Department Rice University Houston, TX United States; 2 Menninger Department of Psychiatry Baylor College of Medicine Houston, TX United States

**Keywords:** SOLVD-Teen and SOLVD-Parent App, adolescent depression, smartphone monitoring, self-evaluation, parental input, sensory data

## Abstract

**Background:**

Depression carries significant financial, medical, and emotional burden on modern society. Various proof-of-concept studies have highlighted how apps can link dynamic mental health status changes to fluctuations in smartphone usage in adult patients with major depressive disorder (MDD). However, the use of such apps to monitor adolescents remains a challenge.

**Objective:**

This study aimed to investigate whether smartphone apps are useful in evaluating and monitoring depression symptoms in a clinically depressed adolescent population compared with the following gold-standard clinical psychometric instruments: Patient Health Questionnaire (PHQ-9), Hamilton Rating Scale for Depression (HAM-D), and Hamilton Anxiety Rating Scale (HAM-A).

**Methods:**

We recruited 13 families with adolescent patients diagnosed with MDD with or without comorbid anxiety disorder. Over an 8-week period, daily self-reported moods and smartphone sensor data were collected by using the *Smartphone- and OnLine usage–based eValuation for Depression* (SOLVD) app. The evaluations from teens’ parents were also collected. Baseline depression and anxiety symptoms were measured biweekly using PHQ-9, HAM-D, and HAM-A.

**Results:**

We observed a significant correlation between the self-evaluated mood averaged over a 2-week period and the biweekly psychometric scores from PHQ-9, HAM-D, and HAM-A (0.45≤|r|≤0.63; *P*=.009, *P*=.01, and *P*=.003, respectively). The daily steps taken, SMS frequency, and average call duration were also highly correlated with clinical scores (0.44≤|r|≤0.72; all *P*<.05). By combining self-evaluations and smartphone sensor data of the teens, we could predict the PHQ-9 score with an accuracy of 88% (23.77/27). When adding the evaluations from the teens’ parents, the prediction accuracy was further increased to 90% (24.35/27).

**Conclusions:**

Smartphone apps such as SOLVD represent a useful way to monitor depressive symptoms in clinically depressed adolescents, and these apps correlate well with current gold-standard psychometric instruments. This is a first study of its kind that was conducted on the adolescent population, and it included inputs from both teens and their parents as observers. The results are preliminary because of the small sample size, and we plan to expand the study to a larger population.

## Introduction

### Background

Depression has a global lifetime prevalence of 28.8% and carries significant financial, medical, and emotional burden on modern society [1,2]. The World Health Organization (WHO) estimates that depression alone accounts for 4.3% of the global burden of disease and is the largest single cause of disability worldwide (according to the WHO Action Plan 2013-2020 [3]). However, a key challenge in the care of persons with depression is that there is often no quantified information about the psychological status of the individual between sporadic clinical psychometric evaluations every few months. Thus, the dynamic changes in an individual’s mental health status remain invisible to health care systems.

Several proof-of-concept studies have highlighted activity changes captured by smartphone sensors, and the usage patterns are associated with depressive symptoms in adult patients with major depressive disorder (MDD) [4,5]. Higher degrees of depression are linked to reduced contact with one’s social network, shorter text messages, reduced duration of calls, and less variability in geospatial activity [6-9]. The same observations were reproduced in younger adults (aged 19-30 years) [10], showing that changes in daily stress levels in young adults aged 19 to 30 years were associated with changes in geospatial activity, sleep duration (obtained by proxy from phone sensors), and sensor-derived speech duration. A pioneer study was conducted by co-designed smartphone apps to monitor mood symptoms for the youth with major depression, suicidal ideation, and self-harm [11]. However, to date, studies on smartphone-based depression monitoring are still limited in the context of teenagers.

### Objective and Results

In this paper, we report the results from a *Smartphone- and OnLine usage–based eValuation for Depression* (SOLVD)-Teen trial with the aim to quantify the use of smartphones in monitoring depression in a clinically depressed adolescent population. We had two main hypotheses. First, we hypothesized that a combination of smartphone sensors’ usage and patient self-reports of daily mood can be predictive of depressive states when compared with standard clinical psychometric instruments such as Patient Health Questionnaire-9 (PHQ-9), Hamilton Rating Scale for Depression (HAM-D), and Hamilton Anxiety Rating Scale (HAM-A). Second, parental reports about the teen’s mood can serve as an important dimension in tracking and predicting the teen’s depressive status as the parents are often involved in the care of their teen and hence can potentially serve as a human *sensor* of their teen’s mood.

We found an affirmative answer to both our hypotheses. Specifically, our key findings are as follows. First, smartphone sensors (GPS and step count) provide coarse information about user mobility, and smartphone usage (phone calls and text messages) provides coarse information about communication patterns. When the above information is combined with daily user self-reports on mood, it can be used to accurately predict future psychometric scores; the details are presented in the Results section. Second, parental reports about a teen’s mood are correlated with the teen’s baseline psychometric scores, and thus, they provide a new dimension for tracking a teen’s mental status. This is an important finding, especially when the teen is not compliant to recording their mood status, and it could provide a new dimension in clinical care by recruiting parents. Third, if we combine parental reports with the teen’s smartphone sensor and usage data, then PHQ-9 can be predicted even more accurately. Thus, parental data provide new *useful* information and make the predictors more accurate. It should be noted that none of our methods rely on the content of messages or calls but only aggregated statistics, for example, frequencies and durations.

### Related Work

Although our results are a promising step toward developing new tools and methods for managing adolescent conditions, the usage of apps to monitor adolescents remains a challenge. Even though teenagers have high rates of smartphone use, they also represent a vulnerable population with a high degree of impulsivity and frequent reports of suicidal ideation; these factors may actually lead to exclusion of more than half depressed teenagers from traditional pharmaceutical trials [12]. In a nationally representative sample of adolescents in the United States, about 22% of teenagers had a mental illness with significant distress, and 40% of those meeting the criteria for one disorder also met the criteria for another class of disorders (eg, anxiety and mood) [1]. Furthermore, teenage years represent the time when many mental disorders start; therefore, a lack of diagnostic clarity is noted in many clinical cases. The onset of anxiety and depression with some mood lability may indicate bipolar disorder [13], and there is a very high comorbidity between anxiety and depressive symptoms in childhood, adolescent, and early adulthood phases [14]. The diagnostic dilemmas of adolescent psychiatry also include the question of whether suicidal ideation due to antidepressant use is a result of subsyndromal undiagnosed bipolar disorder [15].

It should be noted that the treatment of anxiety and depression in adolescents is crucial to ensure healthy life trajectories. In a meta-analysis of studies encompassing more than 762,000 patients, the presence of a childhood or an adolescent mental health disorder significantly increased the odds of alcohol and substance use disorders later in life [16]. The Finnish Health 2011 longitudinal study [17] also shows that young adults with mental health disorders, especially those diagnosed before adulthood, have poorer quality of life and need more support to achieve academic goals.

To date, several apps are available for download, but they have not undergone rigorous research or clinical evaluations [18]. Grist et al [19], in a survey of 775 adolescent girls, found that the use of mental health apps occurred infrequently, no more than 15%, reinforcing that although adolescents are avid technology users, directed use for mental health improvement has not been tested or widely publicized. Thus, it is important to find ways that do *not* require constant user engagement. Toward that end, the SOLVD-Teen approach of passive logging of sensors and usage from teens’ phones and active input from parents could provide a new way of leveraging smartphones, especially passive sensor data.

## Methods

### Smartphone- and OnLine Usage–Based eValuation for Depression App Design

We designed the SOLVD apps with the aim of converting a smartphone to a quantitative *mental health sensor*. The design goal was to have a simple user interface while collecting context-rich sensory data automatically in the background and protecting user privacy. As we are not allowed to continuously track user location and log smartphone usage data in the background on the Apple iOS platform, the SOLVD apps only support Android smartphones.

**Figure 1:**
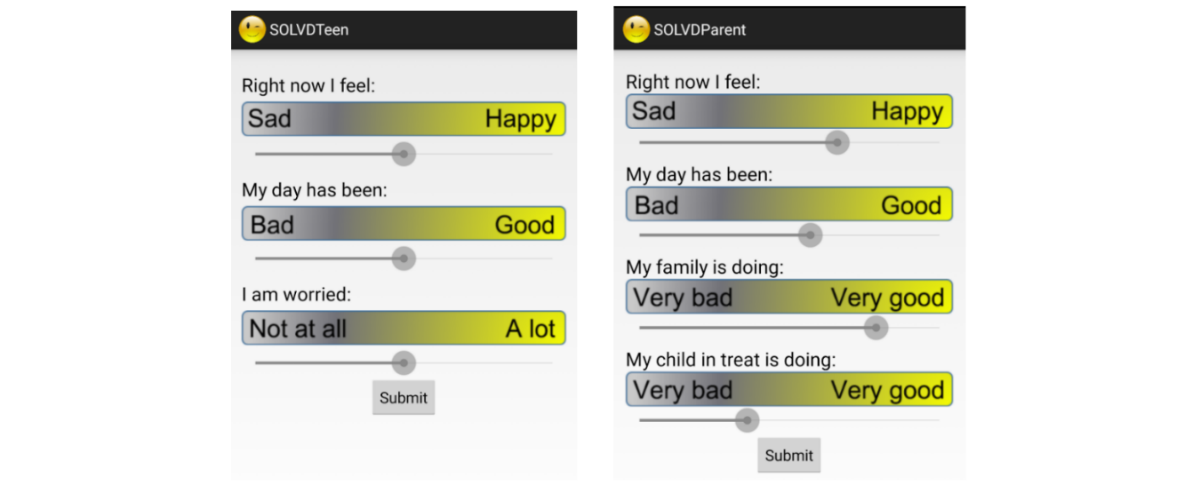
Screenshots of the *Smartphone- and OnLine usage–based eValuation for Depression* (SOLVD)-Teen (left) and SOLVD-Parent (right) apps. The user input is converted to a numerical score between 0 and 100. The frontend of the SOLVD-Parent app is modified to capture both the parents’ self-evaluations and their perceived well-being of their kids.

The SOLVD apps that were targeted at the adolescent population consisted of 2 pairing apps: the SOLVD-Teen app and the SOLVD-Parent app. Both apps have a user-facing module that collects users’ active responses to ecological momentary assessment (EMA) questions, and a background mobile logger that records smartphone sensor and usage data. The user-facing module sends out a notification daily around 8 PM to collect users’ self-evaluations of their mood and anxiety level. The users submit their responses by sliding a bar, and the results are converted to a numerical score between 0 and 100. Meanwhile, the Mobile Logger continuously runs in the background and collects sensor and usage data, including accelerometer, GPS, steps, call log, text messages, screen on and off status, and ambient light intensity. The data are first stored locally and then automatically uploaded to a remote server when the phone is in an idle state and the Wi-Fi is connected. To protect user privacy, we used one-way MD5 hashing on the fly during the data-logging stage to encrypt sensitive information such as phone numbers and email addresses, thus guaranteeing that the user identity is never recorded either locally or remotely. Figure 1 shows the screenshots of the SOLVD-Teen and SOLVD-Parent apps.

### Study Design

The SOLVD-Teen study was an extension of the SOLVD pilot trial [9], which established a correlation between clinician-rated measures, self-rated measures, and sensor-based app measures for depressed adult patients. The SOLVD-Teen trial was designed for adolescent patients together with at least one parent. Each subject or family was required to enroll in the study for 8 weeks and use the SOLVD-Teen app to evaluate the utility of smartphones for the evaluation and monitoring of MDD. The study was approved by the institutional review boards (IRB) of Baylor College of Medicine, Rice University, and Harris Health Systems.

**Figure 2:**
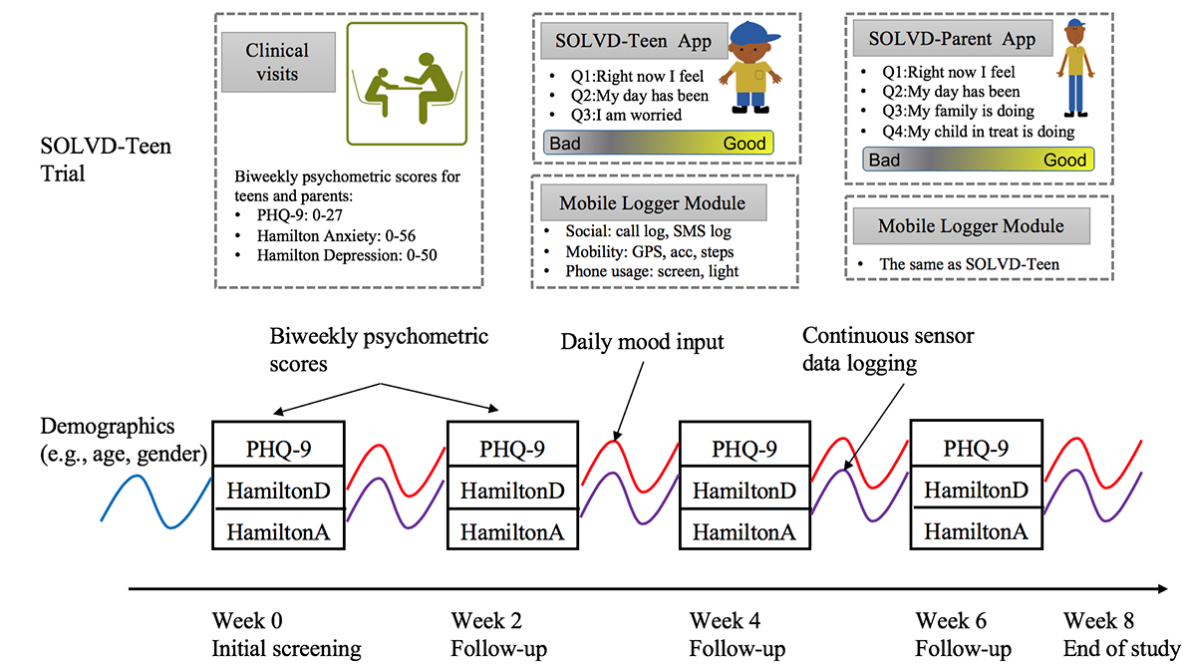
Study design, data collection protocol, and study timeline for the *Smartphone- and OnLine usage–based eValuation for Depression* (SOLVD)-Teen trial. Both SOLVD-Teen and SOLVD-Parent apps have a user-facing module and a background mobile logger. Each subject enrolled in the study for 8 weeks.

We collected 3 forms of data: (1) biweekly in-clinic psychometric scores from PHQ-9, HAM-A, and HAM-D; (2) daily self-inputs submitted via the SOLVD app; (3) smartphone sensor and usage data, including GPS, steps, accelerometer, call log, text messages, screen status, and ambient light intensity, by running an Android logger continuously in the background. For the teen trial, we expanded the data collection protocol and included the teens’ parents in the study. Their parents used the SOLVD-Parent app to submit their self-inputs and log smartphone background data, and they also attended the biweekly clinical assessment. Figure 2 details the data collection protocol and the timeline for the trial.

During the initial visit, the subjects signed their informed consent forms after an explanation of how their phone and sensor data would be collected, stored, and deidentified. Subjects also completed the Mini International Neuropsychiatric Interview (MINI) to confirm a diagnosis of MDD. Standard clinical psychometric instruments such as PHQ-9, HAM-D, and HAM-A were administered to ascertain baseline depression and anxiety symptoms. Subjects were directed to download the SOLVD-Teen or SOLVD-Parent apps on their personal smartphones with the assistance of a research clinician.

For the following weeks, all subjects utilized their personal smartphones to run the SOLVD apps and received standard outpatient treatment as usual over the 8-week duration of the study. During this period, patients were also evaluated biweekly by a research clinician to assess for depression and anxiety symptoms using the HAM-D, HAM-A, and PHQ-9 scales. The clinician was blinded to the smartphone data. The patients were compensated for each of these 4 visits.

### Recruitment

The subjects for the SOLVD-Teen trial were recruited from the outpatient psychiatry clinic at Ben Taub Hospital, Harris Health System, in Houston, Texas, between December 2016 and February 2017. The subjects were recruited from an intensive therapy program, *AIM,* an 8-week intensive individual and group therapy with the patients with depression and their parents. The inclusion criteria included both English- and Spanish-speaking adolescent patients aged 12 to 17 years of both genders with the diagnosis of MDD as confirmed by the MINI. The exclusion criteria included patients with substance use, bipolar disorder, severe conduct disorder, and autism spectrum disorders. All patients were receiving pharmacological treatment at the time of the study, and no medication regimen changes were made through the duration. The study was approved by the IRB of Rice University (IRB number FY2017-241) and Baylor College of Medicine (IRB number H-36157).

### Feature Extraction and Statistical Analysis

The sensor data generated by the SOLVD apps varied in sampling rate and duty cycle. To correlate with the biweekly psychometric scores, we extracted various features on a daily basis, which were related to mobility, social interactions, and daily living context. The details for each sensor type and the extracted features are explained below.

#### Mobility

To understand the association between depressive symptoms and the mobility of the subjects, we had configured the SOLVD-Teen app to track both the step count and the GPS location of its user. Then, we extracted multiple features (essentially, statistical summarizations of the data) to capture the activity level and daily trajectory of the subjects. The details are listed below.

#### Step Counter

When each step was detected by the step counter, we recorded the timestamp associated with that step. Then, we computed the total number of steps during nighttime (11 PM-7 AM) and daytime (7 AM-11 PM) per day.

#### GPS Location

**Figure 3:**
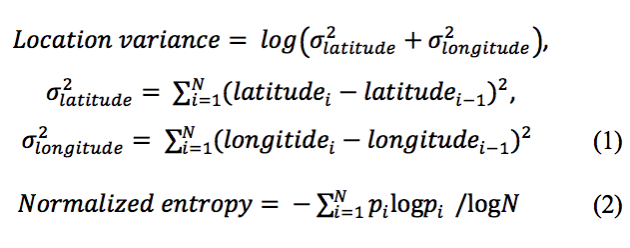
Definition and equations of location variance and normalized entropy.

We logged each GPS data point when the displacement from the last point is larger than 5 m or when the time elapsed from the last point is longer than 5 s. For the data points collected, first, we estimated the moving speed at each location and determined whether each data point belonged to a stationary state (speed<0.2 m/s) or transition state (speed≥1 m/s). Then for data points in stationary states, we used K-means clustering to group all data points into location clusters. The K-means clustering identifies the points around which different data points are clustered, allowing for summarization of the whole data by fewer key points. As we had no prior information regarding the frequently visited places, we started with 1 cluster and gradually increased the number of clusters until all points fell within 500 m from a cluster center. Next, for data points in transitional states, we categorized the transportation modes into *automobile* (7-45 m/s), *walking* (1-2 m/s), and *unknown* based on the moving speed. Finally, we extracted the total distance, transition time, location variance, number of frequently visited places, normalized entropy, and home stay for each subject on a daily basis [20-22]. The speed thresholds are manually chosen, and the definition for each feature is listed below:

1. Total distance, which is defined as the total distance in kilometers traveled by a participant per day, was computed by accumulating the geodesic distance between 2 individual points using the Vincenty formula [23,24].
2. Transition time, which is defined as the duration of each transportation mode (automobile, walking, and unknown) categorized by the moving speed.
3. Location variance, which is defined as the logarithm of sum of the statistical variances of the longitude and latitude for each stationary point, as in Equation (1) in Figure 3. It measures the variability in each subject’s GPS location per day. A higher location variance is related to higher mobility.
4. Number of frequently visited places per day, which is defined as the number of frequently visited places for each day, out of all the frequently visited places identified by the K-means algorithm over the entire study period.
5. Normalized entropy is defined in Equation (2) in Figure 3, where i=1..., N represented a frequently visited place out of the N clusters, and was the percentage of time that the participant spent at place i. It measures the uniformity of the time the participant spent at the frequently visited places each day. The higher the entropy, the more uniformly the participant spent time across different places. Conversely, lower entropy indicates more fraction of the time in a fewer places out of the N clusters.

#### Social Interactions

To study how social interactions are associated with depressive symptoms, we logged the phone calls and text messages of the subjects. Then, we computed different features based on the aggregated statistics to quantify phone-based social interactions, specifically, as follows:

#### Call Log

For each phone call, we logged its timestamp, phone number, type (incoming, outgoing, and missing), and duration. The phone number was encrypted using one-way MD5 hashing to protect user privacy. For each user, we calculated the number of unique phone call partners per day. We also extracted his or her top-10 most frequent contacts during the study period and computed the total number of phone calls, total call duration, and average call duration with the frequent contacts on a daily basis.

#### SMS Log

For each text message, we logged its timestamp, phone number (one-way MD5 hashed), type (incoming or, outgoing), and length in the number of characters. Similar to analyzing phone calls, we also extracted the top 10 most frequent contacts during the study period and computed the total number of text messages, total text message length, and average text message length with the 10 frequent contacts on a daily basis.

#### Daily Living Context

In addition to participant mobility and social interactions, we were also interested in how other daily living contexts were related to depressive symptoms, that is, ambient light intensity that could be an approximation for sleep duration and smartphone screen usage.

#### Light Sensor

The light sensor reading was logged every 2 min. We calculated the average light intensity for each hour. Then, we selected an empirical threshold *τ_I_* for ambient light intensity to separate the scenario of sleeping with the light off from other scenarios. Then, we computed the total number of hours with average light intensity <*τ_I_* during nighttime (11 PM-7 AM) per day, which was used as an indicator for sleep duration.

#### Smartphone Screen Usage

The phone screen status was sampled every 30 seconds. We calculated the percentage of time when the screen was on for each hour, and then computed the number of hours with screen on percentage >*τ_S_* (we empirically selected 10%) during nighttime (11 PM-7 AM) and daytime (7 AM-11 PM) per day.

After completing the feature extraction, we performed the Pearson correlation analysis between each pair of aggregated self-input data and sensor data over 2 weeks and the biweekly psychometric scores and identified those features that were significantly correlated with a depression state for both the adult and the adolescent populations. For the adolescent population with rich sensor data, we also fitted both linear and nonlinear regressors (in particular, support vector regressor with a polynomial kernel) to predict PHQ-9 scores based on self-inputs and sensor data. The reason for fitting a nonlinear regressor is that, a priori, we have no reason to believe that a more accurate predictor of PHQ-9 is linear.

## Results

### Subject Demographics and Adherence

For the SOLVD-Teen trial, we recruited 13 teens diagnosed with MDD from Adolescents in Motion (AIM), an intensive program for teenagers that includes multidisciplinary group therapy with the teenagers and family sessions for the parents. For every teenager recruited, one of their parents was recruited with them. The teens included 11 females and 2 males, with an average age of 14.93 (SD 1.59) years. Out of the 13 subjects, 2 were excluded from further analysis as they dropped out of the study within 2 weeks and did not attend any of the follow-up sessions. The remaining 11 teens had an average PHQ-9 score of 12.72 (SD 5.97), with 8 in the normal-to-mild range (PHQ-9 ≤14) and 3 in the moderate-to-severe range (PHQ-9>14).

Their parents included 11 women and 1 man (there was 1 family with 2 teens) and had an average age of 41.48 (SD 9.75) years. In fact, 5 of the 12 parents were also diagnosed with mild depression and 2 showed moderate-to-severe depression. A total of 8 families completed the entire 8-week trial, and the others enrolled in the study between 4 and 6 weeks. The average teens’ and parents’ compliance to submit daily evaluations through the SOLVD apps was 79.0% (498/630 days) and 95.7% (603/630 days), respectively.

### Relationship Between Smartphone Data and Psychometric Scores

**Figure 4:**
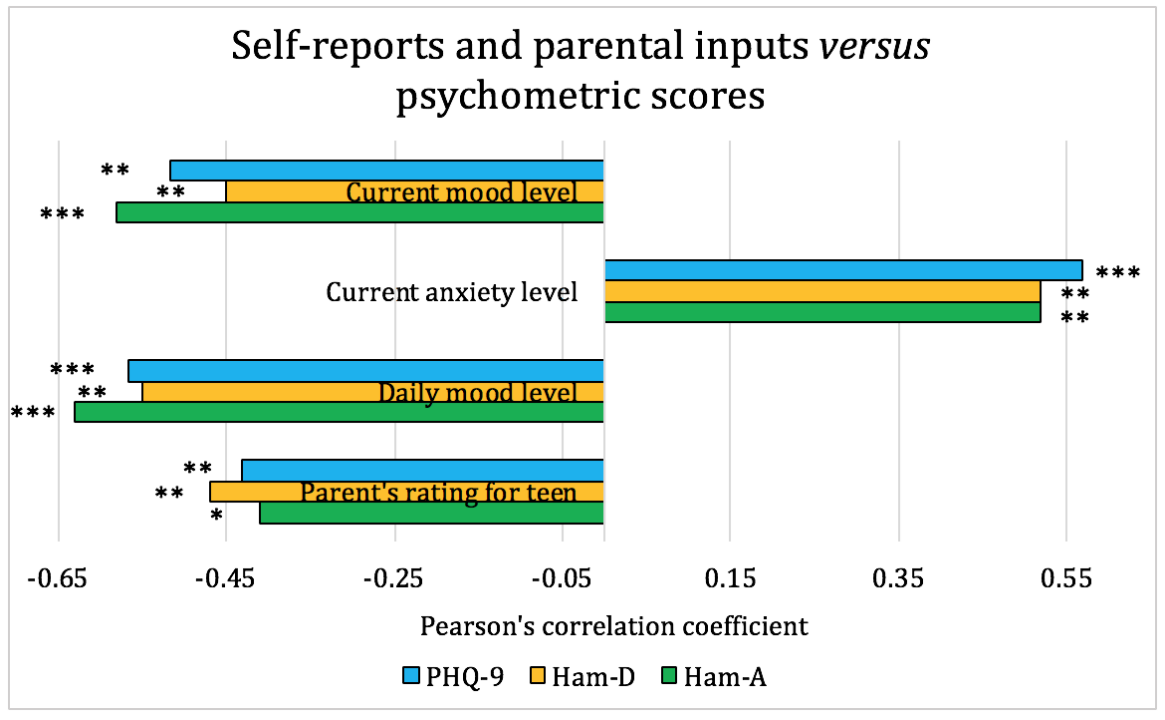
Pearson correlation coefficients between self-reports, parental inputs, and psychometric scores for the adolescent patients. On the basis of the significance level, *P<.1, **P<.05, and ***P<.01. HAM-A: Hamilton Anxiety Rating Scale; HAM-D: Hamilton Rating Scale for Depression; PHQ-9: Patient Health Questionnaire-9.

**Figure 5:**
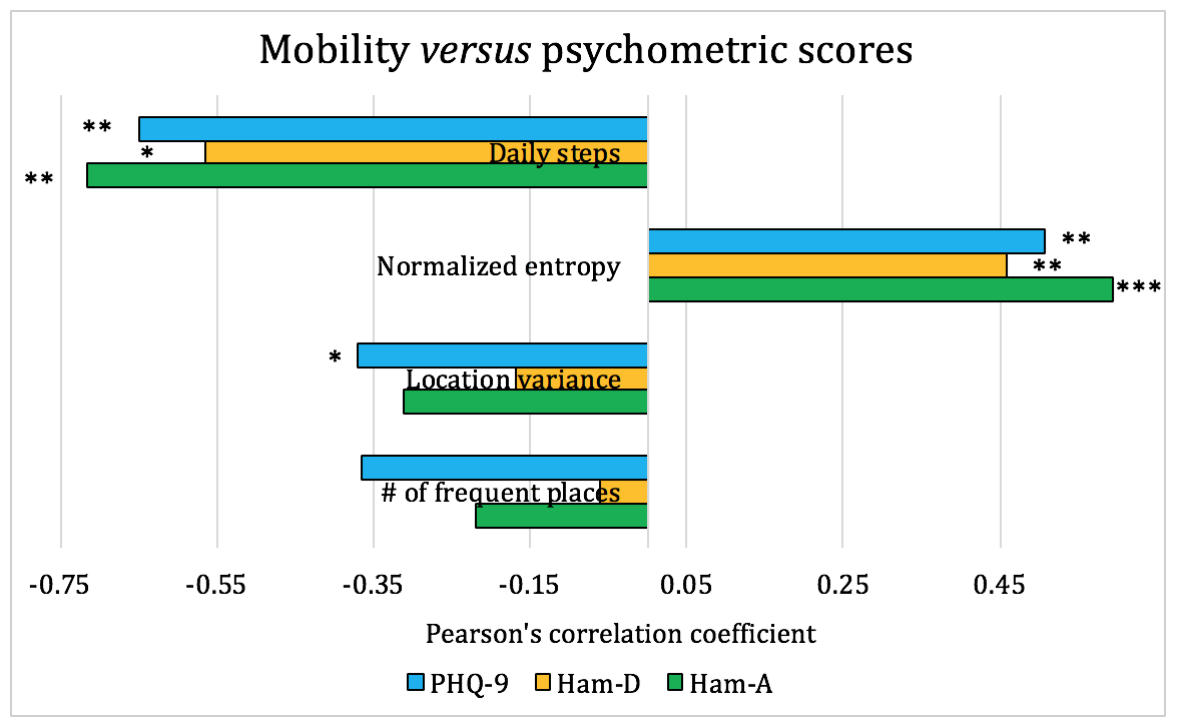
Pearson correlation coefficients between mobility and psychometric scores for the adolescent patients. On the basis of the significance level, *P<.1, **P<.05, and ***P<.01. HAM-A: Hamilton Anxiety Rating Scale; HAM-D: Hamilton Rating Scale for Depression; PHQ-9: Patient Health Questionnaire-9.

**Figure 6:**
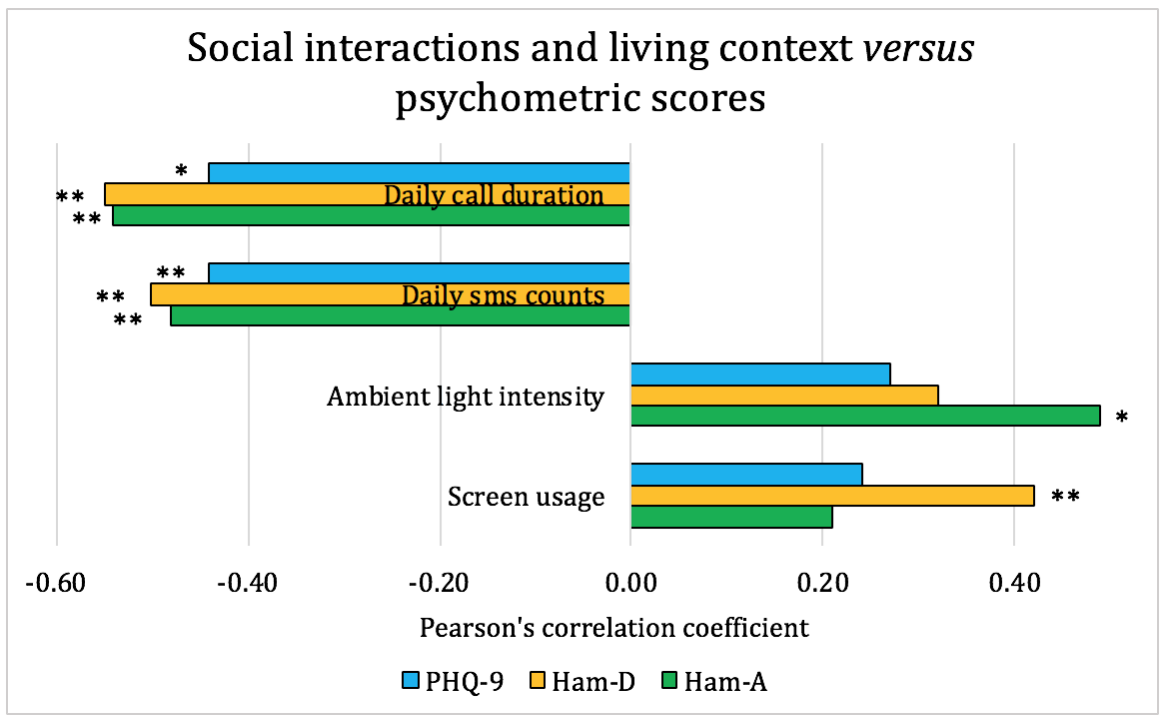
Pearson correlation coefficients between social interactions, living context, and psychometric scores for the adolescent patients. On the basis of the significance level, *P<.1, **P<.05, and ***P<.01. HAM-A: Hamilton Anxiety Rating Scale; HAM-D: Hamilton Rating Scale for Depression; PHQ-9: Patient Health Questionnaire-9.

We used the pair-wise Pearson coefficient as an indicator of the correlations between teens’ self-reports, parental inputs, smartphone sensor data, and clinical psychometric instruments (PHQ-9, HAM-A, and HAM-D). The smartphone sensor data were represented by mobility, social interaction, and living context–related features extracted using methods in Section *Methods-Feature Extraction and Statistical Analysis*. The analysis and the correlations are shown in Figures 4-6.

Figure 4 shows the Pearson correlation coefficient between teens’ self-reports, their parents’ ratings, and the biweekly psychometric scores (PHQ-9, HAM-D, and HAM-A). From Figure 4, we observed that both teens’ and parents’ ratings for mood and anxiety level are significantly correlated with psychometric scores, and teens’ self-reports have a slightly higher correlation than parental inputs. Therefore, daily responses submitted through a customized smartphone app by either teens or their parents can be used as a reliable approach for monitoring depression.

Figure 5 shows the correlations between mobility and psychometric scores, where mobility is captured by steps taken and daily trajectory. From the figure, we observed that subjects with higher depression scores tend to have lower mobility, as indicated by fewer steps taken. Patients with more severe depression also visit fewer places and have lower location variance, yet they spend time more uniformly across different places, as reflected by higher normalized entropy. Similar results were also reported in previous studies with depressed adult patients, thus further validating our observations [25]. Although the overall interpretation matches our understanding that depression generally reduces mobility, higher entropy does not seem to have a clear interpretation, and thus it needs further investigation.

The correlations between social interactions, other living context, and psychometric scores are shown in Figure 6. The results indicate that a higher depression score is significantly correlated with lower social interaction level, such as shorter phone call durations and fewer text messages. Conversely, there is no significant correlation between other living contexts, that is, ambient light intensity, smartphone screen usage, and psychometric scores. Thus, as expected, communication patterns are affected by depression severity, but our dataset did not indicate any significant patterns regarding screen usage and ambient light intensity, with the latter being a proxy for sleep duration.

Overall, the results indicate a strong correlation between daily smartphone-based EMA questions (ie, teens’ self-reports and parental inputs) and clinician-rated psychometric scores. Depressive symptoms assessed by clinical instruments were also significantly correlated with mobility level and social interactions captured by passive smartphone sensor data. However, we did not observe significant correlations between other daily living contexts (ie, ambient light intensity and smartphone screen usage) and depressive symptoms.

### Predicting Depression Severity Using Smartphone Data

We further fitted 2 regression models to predict the psychometric scores from the smartphone data, specifically a linear regressor and a support vector regressor with a polynomial kernel. The linear regressor fits a linear model to the data, whereas the support vector regressor fits a nonlinear model.

To evaluate the model performance in the prediction of PHQ-9 scores, we divided the entire dataset into training and test subsets with a ratio of 4:1. Then, we computed the root mean square error (RMSE) of the predicted PHQ-9 scores on the test set and experimented with different selections of feature subsets to decide which combination gave the highest accuracy. The results are shown in Table 1.

The results indicate that by using either teens’ inputs or parents’ inputs only, we can achieve similar accuracy in PHQ-9 prediction, with the RMSE being 3.38 and 3.47 (the range for PHQ-9 is 0-27), respectively. By using the teens’ self-evaluations, as well as SMS, call, steps, and GPS data, we could predict the PHQ-9 score with an RMSE of 3.23. Furthermore, by adding the parents’ evaluations, we could reduce the RMSE to 2.65, which is also the lowest error that we could achieve. Conversely, if we use only smartphone sensor and usage data, the RMSE is 2.77.

The major significance of the result is that there appears to be significant predictability of clinical measures from smartphone data. Besides, we could achieve similar PHQ-9 prediction accuracy by using only background sensor data, compared with using both active self-reports and passive sensor data, thereby reducing the efforts of manual inputs. Moreover, parents’ inputs provide comparable performance in depression monitoring as teens’ inputs. This clearly shows the potential of mobile-based measurements for tracking depressive states and the importance of introducing someone close to the subjects (eg, their parent) as a *human sensor* to provide an additional dimension of data.

**Table 1:** Root mean square error and variance of predicting Patient Health Questionnaire-9 scores using different subsets of features extracted from smartphone data.

Feature subset	Linear regressor		Support vector regressor	
	RMSE^a^	Variance	RMSE	Variance
All features: both teens’ and parents’ inputs, steps, GPS, SMS, call, light, and screen	9.36	−10.12	3.88	−0.91
Teens’ self-inputs	3.38	−0.45	6.41	−4.21
Parents’ evaluations	3.47	0.15	6.30	−4.03
Both teens’ and parents’ inputs	3.27	−0.36	6.03	−3.61
All sensors: steps, GPS, SMS, call, light, and screen	3.23	−0.32	5.92	−3.44
Steps, GPS, SMS, and call	2.77	0.01	4.77	−1.89
Teens’ inputs, steps, GPS, SMS, and call	10.78	−13.03	3.23	−0.33
Parents’ inputs, steps, GPS, SMS, and call	3.70	−0.73	3.77	−0.80
Teens’ and parents’ inputs, steps, GPS, SMS, and call	8.06	−7.24	2.65^b^	0.11^b^

^a^RMSE: root mean square error.

^b^*P*=.017.

## Discussion

### Principal Findings

The SOLVD-Teen is a first-of-its-kind study that investigated the feasibility of evaluating depressive symptoms of adolescents using smartphone sensor data, daily self-reports, and parental inputs through a customized smartphone app.

The proposed approach worked well for teenagers who are typically heavy smartphone users. Submitting the responses through the app only took less than 15 s per day. Both the teens and their participating parents showed a high adherence rate to submitting daily self-evaluations. All the study participants were comfortable with the use of the technology, and neither the teens nor their parents perceived the app as invasive or burdensome. Besides, the data collection of the study was completely through the smartphone app; hence, no additional sensor or cost was needed. The smartphone app consumes around 5 MB data and 13% battery per day, which is well-acceptable for daily monitoring.

Our study indicated that there were significant correlations between daily self-reported moods, parents’ evaluations, and clinical psychometric scores (PHQ-9, HAM-D, and HAM-A). Lower levels of mobility and fewer social interactions were predictive of higher depression symptoms, which was consistent with a decline in mobility and social communications in individuals with depression. However, other daily living contexts such as light intensity and smartphone screen usage were not significantly correlated with depressive symptoms.

Our study expanded on prior studies by suggesting that introducing other people as *human sensors* could further increase the accuracy in depression monitoring. Given the limited sample size, there is no evidence showing that parents’ evaluations were biased by their own mental health status. The highest accuracy in predicting PHQ-9 was 90%, which was achieved by combining the teens’ and parents’ inputs with SMS/call/steps/GPS data. The prediction accuracy could be improved by adding the evaluations from parents apart from self-inputs from the teens. Our study also showed evidence that by using only passive sensor loggings, we could achieve comparable prediction accuracy by using both sensor loggings and self-inputs. Therefore, the SOLVD-Teen app could further reduce user effort by collecting only passive data, while maintaining a comparable depression monitoring effect.

### Limitations and Future Work

The study conclusion is preliminary given the relatively small sample size of 13 families. Study recruitment ended as the program from which the patients were recruited shut down for logistic reasons as the AIM program closed. In addition, 8 out of the 13 families completed the entire 8-week trial: the program necessitated biweekly attendance, which created a burden (time spent at the program and transportation costs and effort), but the treatment team noted that families tended to drop out once their child did better, and intensive treatment was not felt to be as crucial. We plan to extend the study with the reopening of the AIM program in a different location.

In college-aged students, increased ruminations were found to relate to increasing depressive symptoms [26]. Similarly, nonsuicidal self-injury correlates with self-criticism and feeling criticized by others and usually links to a negative affect of brief durations [27]. Such brief moments of ruminations and self-blame are the epitome of what smartphone apps can help us capture and leverage into treatment opportunities. Thus, the aim of mobile health interventions is to move the treatment of adolescents with mood disorders from reactive to proactive and personalized, thus paving the way to truly individualized treatments [28].

In summary, the SOLVD-Teen study verified the feasibility of using smartphone user inputs and background sensor loggings to monitor adolescent patients with MDD. Its key novelty lies in the recruitment of adolescent depression patients and the introduction of parental evaluations as an additional source of inputs. The study was limited by the relatively small sample size and the constraint of using only Android phones because of the lack of permission to access certain sensors on the iOS platform.

## Abbreviations

AIM: Adolescents in Motion
EMA: ecological momentary assessment
HAM-A: Hamilton Anxiety Rating Scale
HAM-D: Hamilton Rating Scale for Depression
IRB: institutional review board
MDD: major depressive disorder
MINI: Mini International Neuropsychiatric Interview
PHQ-9: Patient Health Questionnaire-9
RMSE: root mean square error
SOLVD: Smartphone- and OnLine usage–based eValuation for Depression
WHO: World Health Organization
